# Structural effect of P278A mutation conferring breast cancer susceptibility in the p53 DNA-binding core domain

**DOI:** 10.1186/1753-6561-6-S6-P50

**Published:** 2012-10-01

**Authors:** Y Suneetha, C Kumaraswamy Naidu

**Affiliations:** 1 Department of Zoology, Sri Venkateswara University, Tirupati, 517502, India

## 

One of the common malignancies faced by women around the world is breast cancer. Risk factors for breast cancer include both genetic and non-genetic. Variants in some of the candidate genes are a common risk factor in breast cancer. These genetic variants associated with breast cancer can be classified as high, moderate or low based on relative risk [1]. Among them, genes that predispose to high risk for breast cancer include *TP53*, *BRCA1*, *BRCA2*, *PTEN*, *STK11* and *CDH1.* A large number of studies have assessed the prognostic and predictive role of TP53 alterations in breast cancer. It is well known that *TP53* is mutated in about 30% of breast cancers [2]. We have analyzed the genetic variation that may alter the expression and function of the *TP53* gene using the sequence-homology-based SIFT tool [3] and a structure-based approach using the PolyPhen-2 server [4]. These two computational approaches showed that rs17849781 (P278A) has a deleterious phenotypic effect conferring to breast cancer. Further, we have analyzed the structural effect of the P278A mutation in the p53 DNA-binding core domain by employing different computational methods.

## References

[B1] Mavaddat N Antoniou AC Easton DF Garcia-Closas M Genetic susceptibility to breast cancer Mol Oncol 2010 4 174 191 10.1016/j.molonc.2010.04.011 20542480 PMC5527934

[B2] Varna M Bousquet G Plassa LF Bertheau P Janin A TP53 status and response to treatment in breast cancers J Biomed Biotechnol 2011 2011 284584 21760703 10.1155/2011/284584 PMC3114547

[B3] Kumar P Henikoff S Ng PC Predicting the effects of coding non-synonymous variants on protein function using the SIFT algorithm Nat Protoc 2009 4 1073 1081 10.1038/nprot.2009.86 19561590

[B4] Adzhubei IA Schmidt S Peshkin L Ramensky VE Gerasimova A Bork P Kondrashov AS Sunyaev SR A method and server for predicting damaging missense mutations Nat Methods 2010 7 248 249 10.1038/nmeth0410-248 20354512 PMC2855889

